# Development and psychometric validation of new questionnaires assessing experienced discrimination and internalised stigma among people with Covid-19

**DOI:** 10.1017/S204579602200021X

**Published:** 2022-05-26

**Authors:** Chiara Bonetto, Davide Pace, Luca Bodini, Morena Colombi, Tine Van Bortel, Antonio Lasalvia

**Affiliations:** 1 Department of Neurosciences, Biomedicine and Movement Sciences, Section of Psychiatry, University of Verona, Verona, Italy; 2 Administrator of the FB Community “Noi che il Covid lo abbiamo sconfitto”, Italy; 3 Leicester School of Allied Health Sciences, Faculty of Health and Life Sciences, De Montfort University, Leicester, UK; 4 Cambridge Public Health Interdisciplinary Research Centre, Department of Psychiatry, University of Cambridge, Cambridge, UK

**Keywords:** Covid-19, discrimination, internalised stigma, psychometric validation

## Abstract

**Aims:**

To develop and validate two new standardised measures assessing, respectively, experienced discrimination (Covid-19 Experienced DISCrimination scale, CEDISC) and internalised stigma (COvid-19 INternalised Stigma scale, COINS) in people who had been infected with severe acute respiratory syndrome coronavirus 2 (SARS-CoV-2) or had developed coronavirus disease 2019 (Covid-19) disease.

**Methods:**

Both the CEDISC and the COINS were developed in Italian and tested for ease of use, comprehension, acceptability, the relevance of items and response options within a focus group session. Online cross-sectional validation survey was conducted among adults infected with SARS-CoV-2 or who developed Covid-19 disease, members of a closed Facebook discussion group in Italy. Exploratory factor analysis (EFA) with Promax oblique rotation; the Kaiser-Meyer-Olkin (KMO) measure of sampling adequacy and the Bartlett's test of sphericity were used to assess the suitability of the sample for factor analysis. Reliability was assessed as internal consistency using Cronbach's alpha and as test–retest reliability using weighted kappa and intraclass correlation coefficient (ICC). Precision was examined by Kendall's tau-b coefficient.

**Results:**

Overall, 579 participants completed the CEDISC, 519 also completed the COINS, 155 completed the retest for both scales after two weeks. The 12 items of the CEDISC converged over a 2-factor solution (‘social life’ and ‘close relations’) accounting for 49.2% of the variance (KMO = 0.894; Bartlett's test *p* < 0.001); the 13 items of the COINS converged over a 3-factor solution (‘self-perception’, ‘close relations’ and ‘social life’) accounting for 67.7% (KMO = 0.827; Bartlett's test *p* < 0.001). Cronbach's *α* was 0.848 for the CEDISC, and 0.837 for the COINS. The CEDISC showed three items (25%) with kappa between 0.61 and 0.80 and seven (58.4%) between 0.41 and 0.60, with only two items scoring 0.21 and 0.40; the COINS had ten items (76.9%) with kappa ranging from 0.41 to 0.60, and three items below 0.31. ICC was 0.906 (95% CI, 0.871–0.932) for the, CEDISC and 0.860 (95% CI, 0.808–0.898) for the COINS. Kendall's tau-b ranged from 0.360 to 0.556 (*p* < 0.001) for the CEDISC and from 0.290 to 0.606 (*p* < 0.001) for the COINS.

**Conclusions:**

Both the CEDISC and the COINS are two valid and reliable scales to be used in studies examining the role of stigma and discrimination of people infected with SARS-CoV-2 and Covid-19 patients, and in research evaluating interventions designed to mitigate stigma in this population.

## Introduction

The worldwide spread of the coronavirus disease 2019 (Covid-19) pandemic has resulted in several psychosocial consequences, including stigmatisation and discriminatory behaviours against people who have, or might have, the disease (Bagcchi, 2020; He *et al*., 2020; Kousoulis *et al*., 2020; WHO, 2020).

Literature on previous viral outbreaks and epidemics reports that infected patients have often been labelled, stereotyped, discriminated against, treated separately and experienced loss of status because of a perceived link with the disease (Van Bortel *et al*., 2016; Baldassarre *et al*., 2020; Lasalvia, 2020; Gronholm *et al*., 2021). Worryingly, it has been found that social stigma often persists even after outbreaks have ended (Overholt *et al*., 2018; James *et al*., 2020).

Social stigma may be experienced by an individual in three forms: enacted stigma (overt behaviours), perceived stigma (awareness of stereotype) and internalised stigma (personal value) (Pescosolido and Martin, 2015). Enacted stigma refers to overt acts of discrimination and humiliation directed at a person because of his or her stigmatised status, which captures the interpersonal aspect of stigma. The process of rejection and unfair treatment experienced by the stigmatised person overlaps with the concept of ‘experienced discrimination’ (Thornicroft *et al*., 2007). By contrast, perceived stigma and internalised stigma capture the intrapersonal aspect of stigma, i.e., perceived stigma refers to the subjective awareness of social stigma, whereas internalised stigma (also known as self-stigma) describes the process of an individual accepting society's negative evaluation and incorporating this into personal value and sense of self (in this process, perceived stigma represents a precondition for the development of internalised stigma).

In the context of the Covid-19 pandemic, experienced discrimination refers to instances during which a given person with suspected or confirmed Covid-19 experiences devaluation, unfair treatment, or exclusion from others due to a perceived link with the disease, whereas internalised stigma refers to the awareness of devaluation or a stereotype of oneself because of a perceived linkage with Covid-19. Due to internalised stigma, people with Covid-19 may discredit themselves and accept that they deserve to be treated unequally and expect to be stigmatised further (Ransing *et al*., 2020). Both experienced discrimination and internalised stigma are interrelated and play a crucial role in the personal and psychological adjustment to the disease. The way stigmatised people respond to stigma - by either conforming to it (self-stigmatisation) or resisting it - can affect the impact of stigma in a community, irrespective of the actual level of enacted stigma or discrimination (Deacon, 2005). High levels of internalised stigma reduce the incentives to challenge stigmatisation, which has negative consequences for both the individual and public health programmes. In fact, in order to avoid discrimination, people infected with severe acute respiratory syndrome coronavirus 2 (SARS-CoV-2) may be reluctant to seek healthcare or may try to hide the disease or misreport symptoms, thus reducing early detection and treatment (Des Jarlais *et al*., 2006; Stangl *et al*., 2019) and posing difficulties in controlling the spread of the infection (Van Bortel *et al*., 2016). Social stigma may also affect the mental health of stigmatised people. Initial evidence suggests that stigma is associated with PTSD symptoms, depression, and anxiety among patients hospitalised with Covid-19 (Liu *et al*., 2020). The experience of being treated differently from others due to Covid-19 may be also indirectly associated with anxiety, depression and insomnia through the mediating effect of shame and internalised stigma (Li *et al*., 2020*a*).

However, the issue of Covid-19 related stigma and its relationships with mental health outcomes in people infected with SARS-CoV-2 has not been sufficiently addressed in the literature and empirical evidence on the extent and severity of stigmatisation among persons surviving Covid-19 is still lacking. This research gap is substantially due to the lack of standardised measures specifically designed to measure interpersonal and intrapersonal aspects of stigma in people with Covid-19. Recently, a measure adapted for Covid-19 patients and based on a previous HIV/AIDS stigma scale has been published. However, this scale does not specifically address the different components of interpersonal (experienced discrimination) and intrapersonal stigma (internalised) (Huang *et al*., 2022) and it seems to lack a sound theoretical basis.

The present paper aims to fill this research gap by reporting on the development and psychometric properties testing of two standardised multidimensional measures assessing, respectively, experienced discrimination and internalised stigma in persons infected with SARS-CoV-2 or who have had full blown Covid-19 disease.

## Methods

### Item generation and pre-testing

A two-phase process was carried out for the development of the scales. In the first phase (February–March 2021), candidate items were identified through a comprehensive literature review of relevant sources addressing personal stigma in patients with infectious diseases (MERS, SARS, Ebola virus and HIV/AIDS). We did not find psychometrically validated scales addressing personal stigma among Covid-19 patients. On the other hand, a series of psychometrically tested scales designed to assess stigma in people with HIV/AIDS were found which represented the basis for item generation in the present study. Specifically, items for the experienced discrimination scale were drawn from Berger *et al*. (2001), whereas for the self-stigma scale were drawn from Sayles *et al*. (2008), which represent the most frequently used measures in the literature for assessing, respectively, HIV related experienced stigma and self-stigma (Earnshaw and Chaudoir, 2009; McAteer *et al*., 2016; Wanjala *et al*., 2021). Moreover, for both experienced discrimination and self-stigma, items were also drawn from Visser *et al*. (2008), who developed three parallel stigma scales to assess personal views of HIV related stigma. With regard the item selection process, for the experienced discrimination scale items were chosen in order to cover all the potential everyday life domains where respondents might have experienced negative reactions from others due to their Covid-19 infection; for the internalised stigma scale, items were chosen in order to cover all the possible negative feelings that respondents might have experienced in relation to their Covid-19 infection or all the public stereotypes related to Covid-19 that respondents might have internalised. In the item generating process, we did not translate any specific item from the original English version of the scales taken into account, but we only extrapolated their thematic elements; therefore, items composing both the newly developed scales were directly produced in Italian. This phase led to the development of two drafted versions of the scales, with one addressing experienced discrimination and the other exploring internalised stigma. Both scales, developed in Italian, were designed as self-administered scales. The two drafted versions contained, respectively, 24 items and 19 items. Respondents indicate their degree of agreement on a 5-point Likert scale ranging from ‘strongly agree’ to ‘strongly disagree’. In the second phase (April 2021), face and content validity of the new scales developed in the previous phase were tested in a focus group session with subjects recovered from Covid-19. This phase aimed to use results obtained from the focus group to decide on final, definitive versions for validation work. Eligible participants for the focus group were identified by the research team. The focus group comprised a purposefully selected sample (Palinkas *et al*., 2015) of nine participants (four women and five men), aged between 25 and 55 years (average age 35,3). They all had developed full blown Covid-19, with two of them having been hospitalised and seven having been treated at home. Participants were asked to discuss aspects of the scales related to ease of use, comprehension, acceptability, relevance of items and response options, time taken to complete and recommendations for improvement. The focus group session took place using an online video conferencing tool and was led by an experienced moderator (A.L.). A previously prepared semi-structured topic guide was followed during the session. The focus group was video recorded (with participant consent) and transcribed for analysis. Based on the learnings and insights from the focus group, minor changes were made to the wording of the items of both questionnaires. Furthermore, in both questionnaires, the various item statements were turned into questions (e.g., from ‘My friends and family members were ashamed of me because I have had Covid-19;’ to ‘How much were your friends and family members ashamed of you because you have had Covid-19?’*)*, responses were categorised using a 4-point Likert scale from ‘not at all’ to ‘a lot’, a ‘Not applicable’ option was added; finally, regarding the experienced discrimination scale items were reduced to 14 and for the internalised stigma scale items were reduced to 17.

### Piloting the questionnaires

The two scales developed according to the steps mentioned above were tested in an online cross-sectional survey conducted between 10 September and 10 November 2021. The newly developed stigma scales were hosted on the web-based survey platform SurveyMonkey and could be self-rated by participants through their PCs, smartphones, or other mobile devices. Participants were adults (⩾18 years) who had been infected with SARS-CoV-2 or had developed Covid-19. The online survey was conducted within a closed Facebook discussion group comprising people who had been infected with SARS-CoV-2 or had developed Covid-19, which at time of the study had more than 10 000 members across Italy. The study description and invitation to participate, as well as the link to the online questionnaires, were posted to all members by the group administrator (M.C.). Participants were aware of the purpose of the study and gave informed consent before participating. They completed the survey anonymously, voluntarily and without any remuneration. To assess test–retest reliability upon completion of all questionnaires, participants were asked to leave their email address if they wished to do so. This subsample was invited to complete the questionnaires again after two weeks.

### The questionnaires

The Covid-19 Experienced DISCrimination scale (CEDISC) and COvid-19 INternalised Stigma scale (COINS), both developed in Italian, were designed to measure, respectively, experienced discrimination and internalised stigma among people infected with SARS-CoV-2 or who had survived Covid-19. The CEDISC starts with an opening question asking participants whether they might have been treated unfairly (e.g., with hostility, avoidance, rejection, prejudice) by other people as they were infected with SARS-CoV-2 or diagnosed with Covid-19 in a series of everyday situations. In order to avoid any possible misunderstanding with the specific content that the scale was aiming to address, a guiding note was given explaining that the situations listed in the questionnaire referred to the period following recovery from Covid-19 (or following isolation due to a positive test), once the respondent had returned to her/his usual social life. The COINS also starts with an opening question asking participants if they might have felt uncomfortable (e.g., embarrassed, ashamed, guilty) as they were infected with SARS-CoV-2 or diagnosed with Covid-19 across a series of possible situations. For both scales, all items are scored on a 4-point Likert scale: 0 = not at all, 1 = a little, 2 = moderately and 3 = a lot. A ‘Not applicable’ option is available. The CEDISC version that was completed in the online survey included 14 questions; however, two items were removed after data analysis as one item had a high number of ‘Not applicable’ responses (36.3%) and the other one had a factor loading <0.40. Thus, the final version of the questionnaire for perceived stigma presented here constitutes 12 items (an English translation of the original Italian scale is shown in Appendix A). The COINS version completed in the online survey included 17 questions, but four items were removed after factor analysis as they had factor loadings <0.40. The final version of the internalised stigma questionnaire therefore comprises 13 items (an English translation of the original Italian scale is shown in Appendix B).

For both questionnaires, a mean score is calculated for the global scale and each subscale. No items are reverse coded. A higher score indicates a higher experienced discrimination and, respectively, internalised stigma level. A strategy for the interpretation of scores may be addressed by applying the midpoint of 1.5, thus identifying four stigma categories: <1 minimal; 1–1.5 low; 1.5–2 moderate; and 2+ high (Brohan *et al*., 2013). In addition, a total score may be calculated by counting the number of items in which individuals scored 1, 2 or 3.

### Statistical analysis

Descriptives were given by absolute frequencies and percentages for categorical variables and means and standard deviations for continuous variables. All analyses were performed with SPSS 26 and Stata 17.

#### Construct validity

Construct validity was established by conducting an exploratory factor analysis (EFA) based on the principal component factoring with Promax rotations. Kaiser-Meyer-Olkin's measure of sampling adequacy (KMO) and Bartlett's test of sphericity were estimated to explore the model's adequacy. Factors with eigenvalues greater than one were retained. Only items with factor loadings >0.4 were considered in the final model. No correlation with other scales measuring similar constructs was performed as no validated instruments assessing experienced discrimination and internalised stigma for Covid-19 were available.

#### Reliability

The reliability was assessed by considering: (1) consistency over subscales (internal consistency) and (2) consistency over time (test–retest reliability). The internal consistency was assessed using Cronbach's *α* with a criterion of 0.70 for a good internal consistency (Cronbach, 1951). To assess test–retest reliability of items, weighted kappa coefficients were calculated with values >0.41 indicating a moderate agreement (Landis and Koch, 1977). Two-way mixed effect intraclass correlation coefficient (ICC) was used to calculate the test–retest reliability for the global mean score and the subscales. A criterion of 0.75 was used to indicate acceptable reliability (Koo and Li, 2016).

#### Precision

The precision (i.e., how well each item fits within the scale) was examined by Kendall's tau-b coefficient. A correlation <0.30 was indicative of an unacceptable fit (Furr, 2021).

#### Acceptability

In order to establish the extent to which the scale was acceptable for the target population, the following aspects were examined: (1) maximum endorsement frequencies (MEF) and (2) aggregate adjacent endorsement frequencies (AEF) (Furr, 2021). In considering MEF, the *n* (%) of respondents endorsing each response category was established. MEF >80% in any category indicates that the item may need further consideration. AEF assesses the proportion of responses in two or more adjacent scale points of an item, where the criterion of >10% was considered acceptable.

#### Feasibility

The feasibility was assessed by registering the time taken to complete each online questionnaire. More than 20 min was considered indicative of an unbearable participant burden. Finally, the percentage of participants who completed each questionnaire was calculated.

## Results

### Participants' characteristics

The online survey involved 579 participants who completed the CEDISC questionnaire, 519 (89.6%) of whom also completed the COINS questionnaire. In terms of socio-demographic and SARS-CoV-2 characteristics, the 60 participants who refused to complete the COINS did not differ from the 519 who completed it, with the only exception of Covid-19 symptoms, which occurred more frequently in people who completed the assessment (93.1% *v* 81.7%, *p* = 0.005 Fisher's exact test). One hundred and fifty-five participants completed the retest after two weeks. No differences were found with respect to socio-demographics and SARS-CoV-2 infection characteristics between the test and the retest samples (Table 1).

**Table 1.** Socio-demographics and clinical characteristics for the test sample (*n* = 579) and the retest sample (*n* = 155)

	Test sample *n* = 579	Retest sample*n* = 155	*p*-Value χ^2^ or Fisher's exact test
Gender, *n* (%)	(33 missing)	(2 missing)	
Male	83 (15.2)	19 (12.4)	0.438
Female	463 (84.8)	134 (87.6)	
Age, *n* (%)			
18–35 years	107 (18.5)	26 (16.8)	0.877
36–55 years	315 (54.4)	87 (56.1)	
56 + years	157 (27.1)	42 (27.1)	
Education, *n* (%)	(1 missing)		
Up to secondary education	115 (19.9)	32 (20.6)	0.971
Tertiary education	253 (43.8)	68 (43.9)	
Degree/postgraduate degree	210 (36.3)	55 (35.5)	
Employment, *n* (%)	(5 missing)		
No	175 (30.5)	44 (28.4)	0.693
Yes	399 (69.5)	111 (71.6)	
Marital status, *n* (%)	(6 missing)		
Single	141 (24.6)	36 (23.4)	0.435
Married/In civil partnership	340 (59.3)	99 (64.3)	
Divorced/widowed	92 (16.1)	19 (12.3)	
Period SARS-CoV-2 infection, *n* (%)			
January–September 2020	111 (19.2)	38 (24.5)	0.170
October–December 2020	223 (38.5)	63 (40.6)	
From January 2021	245 (42.3)	54 (34.8)	
Covid-19 symptoms, *n* (%)			
No	47 (8.1)	8 (5.2)	0.301
Yes	532 (91.9)	147 (94.8)	
Hospitalised for Covid-19, *n* (%)	(47 NA)	(8 NA)	
No	413 (77.6)	114 (77.6)	1.000
Yes	119 (22.4)	33 (22.4)	

### Scoring

The distribution of items pertaining to the final version of both CEDISC and COINS scales are given in Table 2.

**Table 2.** Response frequencies and percentages of the CEDISC (*n* = 579) (top part) and the COINS (*n* = 519) (bottom part)

	Not at all	A little	Moderately	A lot	Not applicable
	*n* (%)	*n* (%)	*n* (%)	*n* (%)	*n* (%)
CEDISC items					
1. Must have done something wrong	286 (49.4)	141 (24.4)	67 (11.6)	49 (8.5)	36 (6.2)
2. Mistake to share with others my Covid	347 (59.9)	97 (16.8)	47 (8.1)	35 (6.1)	53 (9.2)
3. Friends and family ashamed of me	449 (77.5)	65 (11.2)	21 (3.6)	17 (2.9)	27 (4.7)
4. Treated unfairly by family members	467 (80.7)	55 (9.5)	25 (4.3)	18 (3.1)	14 (2.4)
5. Treated unfairly by friends	441 (76.2)	75 (13.0)	27 (4.7)	21 (3.6)	15 (2.6)
6. Treated unfairly in areas of public life	372 (64.2)	79 (13.6)	28 (4.8)	23 (4.0)	77 (13.3)
7. Treated unfairly at work/at school	321 (55.4)	79 (13.6)	41 (7.1)	47 (8.1)	91 (15.7)
8. Treated unfairly by healthcare profess.	230 (39.7)	114 (19.7)	77 (13.3)	136 (23.5)	22 (3.8)
9. Treated unfairly on social media	356 (61.5)	67 (11.6)	25 (4.3)	28 (4.8)	103 (17.8)
10. Media shape negative attitudes	160 (27.6)	71 (12.3)	95 (16.4)	133 (22.6)	122 (21.1)
11. Difficulty returning to work/p. active.	293 (50.6)	110 (19.0)	53 (9.2)	63 (10.9)	60 (10.4)
12. Avoided showing mild resp. symptoms	272 (47.0)	133 (23.0)	71 (12.3)	60 (10.4)	43 (7.4)
COINS items					
1. I am not as good a person as others	325 (62.6)	63 (12.1)	57 (11.0)	60 (11.6)	14 (2.7)
2. I feel ashamed	353 (68.0)	80 (15.4)	43 (8.3)	38 (7.3)	5 (1.0)
3. I feel that it is my fault	258 (49.7)	91 (17.5)	75 (14.5)	94 (18.1)	1 (0.2)
4. I feel embarrassed	227 (43.7)	124 (23.9)	77 (14.8)	86 (16.6)	5 (1.0)
5. Avoid telling others my Covid	328 (63.2)	101 (19.5)	38 (7.3)	38 (7.3)	14 (2.7)
6. Stop socialising for negative reactions	314 (60.5)	86 (16.6)	53 (10.2)	53 (10.2)	13 (2.5)
7. Uncomfortable to go outside of house	241 (46.4)	122 (23.5)	77 (14.8)	73 (14.1)	6 (1.2)
8. Understand if my family avoids me	328 (63.2)	106 (20.4)	45 (8.7)	18 (3.5)	22 (4.2)
9. Understand if friends avoid me	346 (66.7)	96 (18.5)	41 (7.9)	19 (3.7)	17 (3.3)
10. Understand if neighbours avoid me	302 (58.2)	124 (23.9)	41 (7.9)	17 (3.3)	35 (6.7)
11. Agree if employers do not employ me	405 (78.0)	25 (4.8)	8 (1.5)	22 (4.2)	59 (11.4)
12. Understand exclusion from public life	364 (70.1)	44 (8.5)	15 (2.9)	18 (3.5)	78 (15.0)
13. Understand unavailability of doctors	435 (83.8)	37 (7.1)	17 (3.3)	21 (4.0)	9 (1.7)

### Construct validity

By considering the CEDISC scale, 12 items converged over a 2-factor solution accounting for 49.2% of the variance (KMO 0.894; Bartlett's test *p* < 0.001). The first factor, namely ‘Social life’, accounted for 39.7% of the variance and constituted 7 items, while the second factor, namely ‘Close relations’, accounted for 9.5% of the variance and constituted 5 items. Regarding the COINS questionnaire, 13 items converged over a 3-factor solution accounting for a variance of 67.7% (KMO 0.827; Bartlett's test *p* < 0.001). The first factor, namely ‘Self-perception’, accounted for 34.5% of the variance and comprised 7 items. The second factor, namely ‘Close relations’, accounted for 22.7% of the variance and included 4 items. Finally, the third factor, namely ‘Social life’, accounted for 10.5% of the variance and consisted of 3 items (Table 3).

**Table 3.** Factor loadings from the exploratory factor analysis [principal component extraction; Promax rotations; factor loadings >0.4 (in bold) were retained] for the CEDISC (*n*  = 579) (top part) and the COINS (*n* = 519) (bottom part)

	Factor 1	Factor 2		
CEDISC items	Social life	Close relations	–	Communalities
1. Must have done something wrong	0.120	**0.602**		0.460
2. Mistake to share with others my Covid	0.203	**0**.**586**		0.522
3. Friends and family ashamed of me	−0.104	**0**.**895**		0.704
4. Treated unfairly by family members	−0.192	**0**.**847**		0.566
5. Treated unfairly by friends	0.359	**0**.**468**		0.544
6. Treated unfairly in areas of public life	**0**.**597**	0.174		0.508
7. Treated unfairly at work/at school	**0**.**626**	0.134		0.507
8. Treated unfairly by healthcare profess.	**0**.**453**	0.068		0.245
9. Treated unfairly on social media	**0**.**732**	−0.078		0.476
10. Media shape negative attitudes	**0**.**694**	−0.024		0.463
11. Difficulty returning to work/p. active.	**0**.**818**	−0.218		0.509
12. Avoided showing mild resp. symptoms	**0**.**578**	0.085		0.398
Eigenvalue	4.8	1.1		
% Variance explained	39.7%	9.5%		

	Factor 1	Factor 2	Factor 3	
COINS items	Self-perception	Close relations	Social life	Communalities
1. I am not as good a person as others	**0**.**732**	0.006	−0.046	0.528
2. I feel ashamed	**0**.**840**	−0.124	0.041	0.692
3. I feel that it is my fault	**0**.**627**	−0.047	0.107	0.413
4. I feel embarrassed	**0**.**804**	−0.021	0.040	0.652
5. Avoid telling others my Covid	**0**.**678**	−0.106	0.049	0.453
6. Stop socialising for negative reactions	**0**.**720**	0.223	−0.122	0.589
7. Uncomfortable to go outside of house	**0**.**715**	0.085	−0.064	0.526
8. Understand if my family avoids me	−0.025	**0**.**930**	0.016	0.870
9. Understand if friends avoid me	0.025	**0**.**918**	0.039	0.884
10. Understand if neighbours avoid me	−0.026	**0**.**892**	0.003	0.790
11. Agree if employers do not employ me	−0.023	−0.007	**0**.**915**	0.826
12. Understand exclusion from public life	0.036	0.099	**0**.**848**	0.815
13. Understand unavailability of doctors	0.007	−0.020	**0**.**878**	0.759
Eigenvalue	4.5	3.0	1.4	
% Variance explained	34.5%	22.7%	10.5%	

### Reliability

By considering the CEDISC questionnaire, the Cronbach's *α* value for the global score was 0.848 indicating a good internal consistency. The alpha value for the items ranged from 0.831 to 0.850. By considering the two subscales, the Cronbach's alpha was 0.770 and 0.777, respectively. Regarding the COINS questionnaire, the Cronbach's *α* value for the global score was 0.837 indicating a good internal consistency. The alpha value for the items ranged from 0.815 to 0.832. By considering the three subscales, the Cronbach's *α* was 0.855, 0.924 and 0.868, respectively (Table 4).

**Table 4.** Internal consistency for the global score and the subscales (Cronbach's *α*) for the CEDISC (*n* = 579) (top part) and the COINS (*n* = 519) (bottom part)

CEDISC items	Cronbach's *α*			
	Global score	Social life	Close relations	–
1. Must have done something wrong	0.836		0.758	
2. Mistake to share with others my Covid	0.832		0.739	
3. Friends and family ashamed of me	0.836		0.709	
4. Treated unfairly by family members	0.842		0.756	
5. Treated unfairly by friends	0.832		0.720	
6. Treated unfairly in areas of public life	0.833	0.735		
7. Treated unfairly at work/at school	0.831	0.730		
8. Treated unfairly by healthcare profess.	0.850	0.772		
9. Treated unfairly on social media	0.836	0.734		
10. Media shape negative attitudes	0.837	0.735		
11. Difficulty returning to work/p. active.	0.840	0.745		
12. Avoided showing mild resp. symptoms	0.837	0.740		
All items	0.848	0.770	0.777	

COINS items	Global score	Self-perception	Close relations	Social life
1. I am not as good a person as others	0.824	0.833		
2. I feel ashamed	0.817	0.821		
3. I feel that it is my fault	0.828	0.855		
4. I feel embarrassed	0.815	0.821		
5. Avoid telling others my Covid	0.827	0.847		
6. Stop socialising for negative reactions	0.817	0.830		
7. Uncomfortable to go outside of house	0.822	0.834		
8. Understand if my family avoids me	0.829		0.878	
9. Understand if friends avoid me	0.826		0.863	
10. Understand if neighbours avoid me	0.830		0.927	
11. Agree if employers do not employ me	0.832			0.780
12. Understand exclusion from public life	0.828			0.790
13. Understand unavailability of doctors	0.832			0.870
All items	0.837	0.855	0.924	0.868

The COINS subscale ‘Close relations’ showed a value exceeding 0.90 due to the presence of one item (‘I would understand if my neighbours avoided me because I have had Covid-19’) with a Cronbach's *α* of 0.927. This item was retained, despite the possible redundancy, because it addresses a relevant aspect of participants' close relations.

By considering test–retest reliability, three items (25%) in the CEDISC questionnaire had kappa values between 0.61 and 0.80 (substantial agreement) and seven (58.4%) between 0.41 and 0.60 (moderate agreement). Only two items showed a fair agreement (values between 0.21 and 0.40). By considering the COINS questionnaire, ten items (76.9%) had a weighted Cohen's kappa value indicating a moderate agreement (0.41–0.60). The three items in the ‘Social life’ subscale showed 86.6%, 75.6% and 83.0% of agreement on the category ‘not at all’, thus generating a test–retest cross-tabulation which is a sparse matrix (online Supplementary Table S1).

Finally, ICC calculated for the CEDISC questionnaire showed that the global mean score and the ‘Close relationships’ subscale had an excellent test–retest reliability, while the ‘Social life’ had a good reliability. Regarding the COINS questionnaire, ICC indicated a good test–retest reliability for the global score and the two subscales ‘Self-perception’ and ‘Close relations’. For the subscale ‘Social life’ the ICC value was low due to the very high agreement on the same category (‘Not at all’) for all the pertaining items (Table 5).

**Table 5.** Test–retest reliability for the global score and the subscales (intraclass correlation coefficient ICC) for the CEDISC (top part) and the COINS (bottom part) (*n* = 155)

	Intraclass correlation coefficient		
	Value	95% CI	Test–retest reliability^a^
CEDISC			
Global score	0.906	0.871–0.932	Excellent
Social role	0.854	0.799–0.893	Good
Close relationships	0.905	0.869–0.931	Excellent
COINS			
Global score	0.860	0.808–0.898	Good
Self-perception	0.885	0.842–0.916	Good
Close relationships	0.730	0.626–0.806	Moderate/good
Social role	0.231^b^	−0.063 to 0.444	–

a 0.51–0.75 moderate; 0.76–0.90 good; 0.91–1 excellent (Koo and Li, 2016).

b The items pertaining to this subscale agree on the category ‘Not at all’ (86.6%, 75.6% and 83.0%, respectively).

### Precision

By considering the CEDISC questionnaire, the Kendall's tau-b coefficients for the global scale ranged from 0.360 to 0.556 (*p* < 0.001), thus indicating that all items fit well with the score of the scale. Moreover, the two subscales showed values ranging from 0.506 to 0.567 and from 0.469 to 0.705, respectively. The COINS questionnaire showed Kendall's tau-b coefficients ranging from 0.290 to 0.606 (*p* < 0.001) for the global scale score (the lowest value 0.290 pertain to one of the three items which constitute the subscale ‘Social life’). The correlations within each subscale were very good (online Supplementary Table S2).

### Acceptability

Regarding the CEDISC questionnaire, the MEF criterion was slightly violated only by one item, with the ‘not at all’ category showing a frequency of 80.7%. The AEF criterion was violated when considering the adjacent categories of ‘moderately’ and ‘a lot’ for five items, ranging from 6.5% to 9.1%. By considering the COINS questionnaire, the MEF criterion was violated by one item on the category ‘not at all’ (83.8%). The AEF criterion was violated when considering the adjacent categories of ‘a little’ and ‘moderately’ for one item (6.3%) and the categories ‘moderately’ and ‘a lot’ for three items (5.7%, 6.4% and 7.3%).

### Feasibility

The mean completion times for the CEDISC and COINS were, respectively, 4.5 min (SD 2; range 2–16) and 3.5 min (SD 2.5; range 1.5–18). All participants completed the CEDISC scale, while 10.4% refused to fill in the COINS scale.

## Discussion

To our knowledge, this is the first study to develop and validate two questionnaires assessing, respectively, experienced discrimination (CEDISC) and internalised stigma (COINS) among people who had been infected by SARS-CoV-2 or who survived Covid-19.

Research so far has developed specific scales measuring public stigma (stereotypes and misconceptions endorsed by the public in relation to Covid-19) (Kantor and Kantor, 2021; Nochaiwong *et al*., 2021) and perceived social stigma (awareness of public stigma or belief that others hold stigmatising thoughts or stereotypes about Covid-19) (Huang *et al*., 2022). As far as we know, specific standardised scales addressing experienced discrimination (actual experiences of being treated unfairly by others) and internalised stigma (internalisation of the negative stereotypes about Covid-19 endorsed by the general population) are still lacking. With specific regard to internalised stigma, the few research published on this topic suggests that this represents a crucial dimension in predicting adverse mental health outcomes (specifically, PTSD, anxiety, depression, demoralisation, low self-esteem) in patients surviving Covid-19 (Li *et al*., 2020*a*, 2020*b*; Mahmoudi *et al*., 2021), thus deserving special attention. Since social stigma predicts long-term adverse mental health outcomes in people infected with Covid-19, it is critical for Covid-19 interventions to target stigma in order to both reduce its psychosocial impact on people infected with SARS-CoV-2 or who developed the disease and to remove a key factor that may potentially hamper full recovery in those surviving Covid-19 (Ransing *et al*., 2020). Yet, stigma-reduction interventions tailored around people infected with Covid-19 are non-existent. Thus, there is a need for research to generate knowledge to address Covid-19 related stigma and discrimination.

The psychometric evaluation conducted in this study shows that the 12-item CEDISC, with its 2-factor structure, is a reliable and valid self-report measure for assessing experienced discrimination among people who tested positive for SARS-CoV-2 or survived Covid-19. The factor analysis showed that the CEDISC can adequately measure experienced discrimination as a whole and through the two dimensions of ‘Social life’ and ‘Close relations’.

Similarly, the psychometric properties of the 13-item COINS, with a 3-factor structure, reveal that this questionnaire is a reliable and valid self-report measure for assessing internalised stigma in the same population. In detail, the factor analysis showed that the COINS measures internalised stigma as a whole and through the three dimensions ‘Self-perception’, ‘Close relations’ and ‘Social life’. The reliability analysis revealed a good internal consistency and most of the items showed at least moderate agreement in the test–retest comparison for both scales. The precision demonstrates that all items fit well with the scores in both questionnaires. The acceptability, assessed by MEF and AEF, was slightly violated in a minimal number of items.

Finally, both scales were completed within five minutes by most participants, thus proving to be feasible instruments.

These standardised measures, focusing on interpersonal and intrapersonal aspects of social stigma, will allow to gain a more in-depth knowledge on the psychosocial consequences of the Covid-19 pandemic and to promote more research in this field.

### Strengths and limitations

A major strength of this study lies in the sample size of nearly 600 participants, which allowed us to validate the two scales relying on a statistically robust sample. The second strength relates to the inclusion of people who became positive or were diagnosed with Covid-19 in different pandemic waves, which could have reflected a different pattern of stigmatisation. The third strength pertains to the pre-testing phase, which was conducted by engaging a group of participants representing a wide range of characteristics (age, gender and working status). This study has also some limitations. First, the sample used in developing and validating the two scales may not be representative of the overall population of interest, both in terms of socio-demographic composition and in terms of Covid-19 severity: in fact, female gender in our sample is overrepresented, while elderly people are under-represented. Moreover, symptomatic Covid-19 patients or people admitted to hospital for Covid-19 are probably overrepresented (whereas, e.g., those asymptomatic tested positives are underrepresented). Second, as the study was conducted among members of a social media community, selection bias might have been occurred, thus limiting participation of those who do not regularly access to social media and, in general, of all people affected by ‘digital poverty’ and ‘digital inequality’. Third, both scales were developed in Italian and validated within an Italian sample, thus limiting their use in other cultural and geographical contexts. Fourth, both scales did not include open-ended questions, so the study did not collect qualitative data that might have provided more insights into participants' experiences. Fifth, as with any study that relies on online data collection, biases such as response bias and social desirability bias might have affected the results. Finally, due to the time of data collection, participants tested positive or diagnosed in the early pandemic stages were being asked to recall a period that occurred months earlier; thus, their recollections might not be accurate.

## Conclusions and future research

The present study indicates that both the CEDISC and the COINS represent two valid and reliable measures that may be used in studies examining the role of stigma and discrimination in Covid-19 patients, and in research evaluating interventions designed to mitigate stigma and discrimination in this population. Both scales, therefore, could be incorporated into public health surveys as a part of clinical and intervention research. These newly developed scales were specifically designed for Covid-19, but they might be also used in relation to other (similar) types of infections/pandemics in the future.

The factorial structure of both scales should also be replicated by a Confirmative Factor Analysis in a different sample of people infected with SARS-CoV-2 or with Covid-19. Moreover, future studies are needed on the changing dynamics of stigma in different stages of the pandemic. Finally, further research will be necessary to assess the psychometric properties of both scales in different populations and among people from different cultural backgrounds, as there are relevant differences in attitudes towards illness and experiences of illness, health and stigma across cultures. With this latter regard, a cultural adaptation of the scales will be needed as they were developed within a specific geographical context.

## Data Availability

Data will be available upon reasonable request.

## Supplementary material

For supplementary material accompanying this paper visit https://doi.org/10.1017/S204579602200021X. click here to view supplementary material

## Appendix A

### Covid-19 experienced discrimination scale – CEDISC

This questionnaire asks about situations where you might have been treated unfairly (e.g., with hostility, avoidance, rejection, prejudice) by other people because you were infected by SARS-CoV-2 or diagnosed with Covid-19. NOTE: The situations listed below refer to the period following recovery from illness (or following isolation due to positive test) once you have returned to your usual social life.

Each item has 4 response options. Please read each question carefully and choose one answer for each question. If the answer does not apply to you, please answer ‘Not applicable’.

1. *How much people think you must have done something wrong to deserve getting Covid-19?*
  - ☐ Not at all ☐ A little ☐ Moderately ☐ A lot ☐ Not applicable
2. *How much do you think it was a mistake to share with others that you have had Covid-19 as you have been treated unfairly?*
  - ☐ Not at all ☐ A little ☐ Moderately ☐ A lot ☐ Not applicable
3. *How much your friends and family members were ashamed of you because you have had Covid-19?*
  - ☐ Not at all ☐ A little ☐ Moderately ☐ A lot ☐ Not applicable
4. *How much have you been treated unfairly by your family members because you have had Covid-19?*
  - ☐ Not at all ☐ A little ☐ Moderately ☐ A lot ☐ Not applicable
5. *How much have you been treated unfairly by your friends because you have had Covid-19?*
  - ☐ Not at all ☐ A little ☐ Moderately ☐ A lot ☐ Not applicable
6. *How much have you been treated unfairly in areas of public life (e.g., sporting centres, clubs, cultural events, voluntary groups) because you have had Covid-19?*
  - ☐ Not at all ☐ A little ☐ Moderately ☐ A lot ☐ Not applicable
7. *How much have you been treated unfairly at work/at school because you have had Covid-19?*
  - ☐ Not at all ☐ A little ☐ Moderately ☐ A lot ☐ Not applicable
8. *How much have you been treated unfairly by healthcare professionals (GP, nurses, treating clinicians) because you have had Covid-19?*
  - ☐ Not at all ☐ A little ☐ Moderately ☐ A lot ☐ Not applicable
9. *How much have you been treated unfairly on your social media because you have had Covid-19?*
  - ☐ Not at all ☐ A little ☐ Moderately ☐ A lot ☐ Not applicable
10. *Do you think that unfair treatment towards you might have been also determined by mass media information on Covid-19?*
  - ☐ Not at all ☐ A little ☐ Moderately ☐ A lot ☐ Not applicable
11. *How much difficulty have you had returning back to work or other public activities after being tested negative for Covid-19?*
  - ☐ Not at all ☐ A little ☐ Moderately ☐ A lot ☐ Not applicable
12. *How much have you been avoided while showing mild upper respiratory symptoms (e.g., runny nose, coughing, sneezing, etc.) even after being tested negative for Covid-19?*
  - ☐ Not at all ☐ A little ☐ Moderately ☐ A lot ☐ Not applicable

## Appendix B

### Covid-19 internalised stigma scale – COINS

This questionnaire asks about times when you might have felt uncomfortable (e.g., embarrassed, ashamed, guilty) because you were infected by SARS-CoV-2 or diagnosed with Covid-19.

Each item has 4 response options. Please read each question carefully and choose one answer for each question. If the answer does not apply to you, please answer ‘not applicable’.

1. *How much do you feel you are not as good a person as others because you have had Covid-19?*
  - ☐ Not at all ☐ A little ☐ Moderately ☐ A lot ☐ Not applicable
2. *How much do you feel ashamed that you have had Covid-19?*
  - ☐ Not at all ☐ A little ☐ Moderately ☐ A lot ☐ Not applicable
3. *How much do you feel that it is your fault that you have had Covid-19?*
  - ☐ Not at all ☐ A little ☐ Moderately ☐ A lot ☐ Not applicable
4. *How much do you feel embarrassed that you have had Covid-19?*
  - ☐ Not at all ☐ A little ☐ Moderately ☐ A lot ☐ Not applicable
5. *How much have you avoided telling others that you have had Covid-19?*
  - ☐ Not at all ☐ A little ☐ Moderately ☐ A lot ☐ Not applicable
6. *How much have you stopped socialising with people because of their negative reactions to your having Covid-19?*
  - ☐ Not at all ☐ A little ☐ Moderately ☐ A lot ☐ Not applicable
7. *How much do you feel uncomfortable to go anywhere outside of my house because you have had Covid-19?*
  - ☐ Not at all ☐ A little ☐ Moderately ☐ A lot ☐ Not applicable
8. *How much would you understand your family members if they avoided you because you have had Covid-19?*
  - ☐ Not at all ☐ A little ☐ Moderately ☐ A lot ☐ Not applicable
9. *How much would you understand friends if they avoided you because you have had Covid-19?*
  - ☐ Not at all ☐ A little ☐ Moderately ☐ A lot ☐ Not applicable
10. *How much would you understand your neighbours if they avoided you because you have had Covid-19?*
  - ☐ Not at all ☐ A little ☐ Moderately ☐ A lot ☐ Not applicable
11. *How much would you agree with employers if they would not employ you because you have had Covid-19?*
  - ☐ Not at all ☐ A little ☐ Moderately ☐ A lot ☐ Not applicable
12. *How much would you understand people if they excluded you from areas of public life (e.g. sporting centres, clubs, cultural events, voluntary groups) because you have had Covid-19?*
  - ☐ Not at all ☐ A little ☐ Moderately ☐ A lot ☐ Not applicable
13. *How much would you understand doctor if he/she was not available to see you because you have had Covid-19?*
  - ☐ Not at all ☐ A little ☐ Moderately ☐ A lot ☐ Not applicable
