# Supplementary material for: Development and psychometric validation of new questionnaires assessing experienced discrimination and internalised stigma among people with Covid-19
Source: Epidemiol Psychiatr Sci. 2022 May 26;31:e37. doi: 10.1017/S204579602200021X (PMC9158394; doi:10.1017/S204579602200021X)
Supplement: Supplementary file 1 [file S204579602200021Xsup001.docx]

**On-line Supplementary Material**

**Table 1.** Items’ test-retest reliability (Weighted Cohen’s kappa) for the CEDISC (top part) and the COINS (bottom part) (n=155)

|  |  | **Weighted Cohen’s kappa** | | |
| --- | --- | --- | --- | --- |
| **CEDISC items** | **Raw % Agreement** | **Value** | **95% CI** | **Agreement^§^** |
| 1 Must have done something wrong | 62.0 | 0.555 | 0.452-0.659 | Moderate |
| 2 Mistake to share with others my Covid | 64.3 | 0.491 | 0.350-0.631 | Moderate |
| 3 Friends and family ashamed of me | 88.4 | 0.676 | 0.519-0.833 | Substantial |
| 4 Treated unfairly by family members | 80.3 | 0.546 | 0.387-0.706 | Moderate |
| 5 Treated unfairly by friends | 77.0 | 0.510 | 0.369-0.651 | Moderate |
| 6 Treated unfairly in areas of public life | 80.5 | 0.680 | 0.547-0.813 | Substantial |
| 7 Treated unfairly at work/at school | 71.6 | 0.659 | 0.547-0.771 | Substantial |
| 8 Treated unfairly by healthcare profess. | 58.4 | 0.575 | 0.477-0.672 | Moderate |
| 9 Treated unfairly on social media | 73.3 | 0.467 | 0.301-0.633 | Moderate |
| 10 Media shape negative attitudes | 40.2 | 0.327 | 0.189-0.466 | Fair |
| 11 Difficulty returning to work/p. active. | 56.1 | 0.390 | 0.251-0.530 | Fair |
| 12 Avoided showing mild resp. symptoms | 56.8 | 0.494 | 0.376-0.612 | Moderate |
| **COINS items** | **Raw % Agreement** | **Value** | **95% CI** | **Agreement^§^** |
| 1 I am not as good a person as others | 69.4% | 0.528 | 0.402-0.654 | Moderate |
| 2 I feel ashamed | 65.4% | 0.514 | 0.404-0.624 | Moderate |
| 3 I feel that it is my fault | 62.1% | 0.583 | 0.483-0.682 | Moderate |
| 4 I feel embarrassed | 46.7% | 0.405 | 0.294-0.516 | Fair/Moderate |
| 5 Avoid telling others my Covid | 65.7% | 0.479 | 0.352-0.606 | Moderate |
| 6 Stop socializing for negative reactions | 65.8% | 0.525 | 0.411-0.640 | Moderate |
| 7 Uncomfortable to go outside of house | 59.7% | 0.556 | 0.461-0.651 | Moderate |
| 8 Understand if my family avoids me | 71.0% | 0.438 | 0.298-0.577 | Moderate |
| 9 Understand if friends avoid me | 70.5% | 0.450 | 0.317-0.584 | Moderate |
| 10 Understand if neighbours avoid me | 67.1% | 0.461 | 0.329-0.592 | Moderate |
| 11 Agree if employers do not employ me | 89.0%^#^ | 0.307 | -0.029-0.543 | - |
| 12 Understand exclusion from public life | 80.5%^#^ | 0.203 | 0.002-0.405 | - |
| 13 Understand unavailability of doctors | 85.7%^#^ | 0.211 | -0.013-0.436 | - |

^§^ 0.21-0.40 fair; 0.41-0.60 moderate; 0.61-0.80 substantial (Landis & Koch, 1977); ^#^ The majority of agreement pertains to the category ‘Not at all’: 86.6% for item 17, 75.6% for item 18 and 83.0% for item 19. The cross-tabulation test vs retest shows a sparse matrix for each item

**Table 2.** Precision for the global score and the subscales (Kendall’s tau-b) for the CEDISC (n=579) (top part) and the COINS (n=519) (bottom part)

|  | **Kendall’s tau-b** | | | |
| --- | --- | --- | --- | --- |
| **CEDISC items** | **Global score** | **Social life** | **Close relations** | **-** |
| 1 Must have done something wrong | 0.469 |  | 0.705 |  |
| 2 Mistake to share with others my Covid | 0.482 |  | 0.609 |  |
| 3 Friends and family ashamed of me | 0.448 |  | 0.551 |  |
| 4 Treated unfairly by family members | 0.360 |  | 0.469 |  |
| 5 Treated unfairly by friends | 0.497 |  | 0.560 |  |
| 6 Treated unfairly in areas of public life | 0.519 | 0.529 |  |  |
| 7 Treated unfairly at work/at school | 0.521 | 0.531 |  |  |
| 8 Treated unfairly by healthcare profess. | 0.461 | 0.520 |  |  |
| 9 Treated unfairly on social media | 0.487 | 0.506 |  |  |
| 10 Media shape negative attitudes | 0.556 | 0.567 |  |  |
| 11 Difficulty returning to work/p. active. | 0.497 | 0.545 |  |  |
| 12 Avoided showing mild resp. symptoms | 0.522 | 0.541 |  |  |
| **COINS items** | **Global score** | **Self-perception** | **Close relations** | **Social role** |
| 1 I am not as good a person as others | 0.500 | 0.585 |  |  |
| 2 I feel ashamed | 0.491 | 0.594 |  |  |
| 3 I feel that it is my fault | 0.450 | 0.523 |  |  |
| 4 I feel embarrassed | 0.606 | 0.670 |  |  |
| 5 Avoid telling others my Covid | 0.415 | 0.485 |  |  |
| 6 Stop socializing for negative reactions | 0.535 | 0.599 |  |  |
| 7 Uncomfortable to go outside of house | 0.530 | 0.619 |  |  |
| 8 Understand if my family avoids me | 0.417 |  | 0.874 |  |
| 9 Understand if friends avoid me | 0.451 |  | 0.851 |  |
| 10 Understand if neighbours avoid me | 0.438 |  | 0.874 |  |
| 11 Agree if employers do not employ me | 0.309 |  |  | 0.744 |
| 12 Understand exclusion from public life | 0.363 |  |  | 0.826 |
| 13 Understand unavailability of doctors | 0.290 |  |  | 0.786 |
